# Association of hearing loss to brain structure and cognition in the Framingham Heart Study

**DOI:** 10.1002/alz70860_101741

**Published:** 2025-12-23

**Authors:** Francis B Kolo, Sophia Lu, Alexa S Beiser, Lily Francis, Debora Melo van Lent, Monica Goss, Saptaparni Ghosh, Shu Jie Ting, Ayantika Banerjee, Timothy Kowalczyk, Nancy Heard‐Costa, Christopher Graham Wood, Pauline Maillard, Evan Fletcher, Charles S. DeCarli, Bradley Duane Welling, Sharon G Kujawa, Sudha Seshadri

**Affiliations:** ^1^ Glenn Biggs Institute, San Antonio, TX, USA; ^2^ Boston University, Boston, MA, USA; ^3^ Boston University School of Medicine, Boston, MA, USA; ^4^ Boston University Chobanian & Avedisian School of Medicine, Boston, MA, USA; ^5^ Glenn Biggs Institute for Alzheimer's & Neurodegenerative Diseases, UT Health San Antonio, San Antonio, TX, USA; ^6^ Glenn Biggs Institute for Alzheimer's & Neurodegenerative Diseases, University of Texas Health San Antonio, San Antonio, TX, USA; ^7^ Glenn Biggs Institute for Alzheimer's & Neurodegenerative Diseases, University of Texas Health Science Center, San Antonio, TX, USA; ^8^ The Framingham Heart Study, Framingham, MA, USA; ^9^ Framingham Heart Study, Framingham, MA, USA; ^10^ Department of Biostatistics, Boston University School of Public Health, Boston, MA, USA; ^11^ Harvard Medical School, Boston, MA, USA; ^12^ Department of Neurology and Center for Neuroscience, University of California, Davis, Davis, CA, USA; ^13^ University of California, Davis, Davis, CA, USA; ^14^ Department of Neurology & Imaging of Dementia and Aging Laboratory, University of California, Davis, Davis, CA, USA; ^15^ Glenn Biggs Institute for Alzheimer's & Neurodegenerative Diseases, University of Texas Health Sciences Center at San Antonio, San Antonio, TX, USA

## Abstract

**Background:**

Age‐related hearing loss is a potentially modifiable risk factor for cognitive impairment and dementia. We related a measure of the severity of hearing loss, the pure tone average (PTA) hearing threshold on audiometry, to measures of brain aging, vascular injury, cognitive function, and incident dementia outcomes.

**Method:**

Framingham Heart Study (FHS) participants (*n* = 1656 [mean age: 58 years]) attending their 6^th^ quadrennial examination, who were free of stroke and dementia, were evaluated for hearing loss (HL), and underwent subsequent brain MRI and cognitive assessment, were studied. PTA for hearing loss was calculated as the average of air conduction thresholds at four frequencies (0.5, 1, 2, 4 kHz). We defined HL as a PTA threshold > 25 dB in the better ear, and we additionally considered four PTA threshold categories (range, in dB HL): Normal [0,16), Slight [16,26), Mild [26‐40), and Moderate or greater (>40) dB. We used multivariate linear regression and related PTA and HL to MRI and cognitive measures, to change in these measures over 4 – 8 years of follow‐up and to incident all‐cause dementia, using Cox proportional hazards regression. The covariates assessed were sex, age, education level, and APOE‐4 status.

**Result:**

At least mild HL was associated with smaller brain volume (*p* = 0.02) by an amount equivalent to 1.75 years of aging at a baseline age of 60 years, and with a decline in executive function on follow‐up (*p* = 0.009]). Over the same time period, higher PTA was associated with increased white matter hyperintensity volume (*p* = 0.048). Slight or greater HL (^3^16 dB) was associated with a higher risk (71%) of developing all‐cause dementia (Hazard Ratio [HR]:1.71, 95% confidence interval [CI]: (1.01, 2.90), *p* = 0.04), over 15 years of follow‐up. This association was driven by participants with at least one APOE‐4 allele (HR:2.86, 95%CI: (1.12, 7.28), *p* = 0.03).

**Conclusion:**

Midlife hearing loss was associated with smaller brain volumes, accelerated decline in executive function, accelerated accrual of white matter abnormalities, and a near doubling of dementia risk, suggesting a potential causal or biomarker role for midlife hearing loss in the prevention of late‐life dementia.

